# Studying signal compartmentation in adult cardiomyocytes

**DOI:** 10.1042/BST20190247

**Published:** 2020-02-27

**Authors:** Aleksandra Judina, Julia Gorelik, Peter T. Wright

**Affiliations:** National Heart and Lung Institute, Faculty of Medicine, Imperial College London, London, U.K.

**Keywords:** calcium, cAMP, cardiovascular, caveolae, compartmentation, t-tubules

## Abstract

Multiple intra-cellular signalling pathways rely on calcium and 3′–5′ cyclic adenosine monophosphate (cAMP) to act as secondary messengers. This is especially true in cardiomyocytes which act as the force-producing units of the cardiac muscle and are required to react rapidly to environmental stimuli. The specificity of functional responses within cardiomyocytes and other cell types is produced by the organellar compartmentation of both calcium and cAMP. In this review, we assess the role of molecular localisation and relative contribution of active and passive processes in producing compartmentation. Active processes comprise the creation and destruction of signals, whereas passive processes comprise the release or sequestration of signals. Cardiomyocytes display a highly articulated membrane structure which displays significant cell-to-cell variability. Special attention is paid to the way in which cell membrane caveolae and the transverse-axial tubule system allow molecular localisation. We explore the effects of cell maturation, pathology and regional differences in the organisation of these processes. The subject of signal compartmentation has had a significant amount of attention within the cardiovascular field and has undergone a revolution over the past two decades. Advances in the area have been driven by molecular imaging using fluorescent dyes and genetically encoded constructs based upon fluorescent proteins. We also explore the use of scanning probe microscopy in the area. These techniques allow the analysis of molecular compartmentation within specific organellar compartments which gives researchers an entirely new perspective.

## Introduction

It is now widely accepted that in order to mediate cellular responses with specificity a secondary messenger response must be spatiotemporally restricted. These ‘regions’ have widely been referred to as microdomains in the literature [1] encapsulating the idea that they occur within a micrometre range. Current data suggests that these domains often operate on the order of nanometres [2].

Intra-cellular signal compartmentation can be defined as the sum of passive or active processes which restrict messenger molecule transit at a sub-organellar level in order to create functional specificity.

These processes define cell type behaviour and even specific cellular sub-types. As the isolated cardiomyocyte is studied as the fundamental unit of force production within the myocardium, these findings may lead to a revision of our understanding of the contribution of these cells to the emergent function of myocardial contractility. In this review we will describe these phenomena, provide a brief guide to the methodologies used to investigate them. In this review, we will almost exclusively focus on the compartmentation of calcium (Ca^2+^) and the secondary messenger 3′–5′-cyclic adenosine monophosphate (cAMP). However, we speculate that these processes are germane within cellular biology and that other signalling processes dependent of the transmission of secondary messenger molecules would be broadly similar and generally amenable to investigation with the techniques that we introduce.

## Organellar basis of cardiomyocyte function

The essential role of an adult cardiomyocyte is to contract rhythmically to allow the production of force and the propulsion of blood [3]. As a result, the cardiomyocytes act as a sophisticated support system for the myofilaments which conduct cellular contraction. The contraction of this protein system is defined by the cyclic modulation of calcium concentrations. Within adult ventricular cardiomyocytes this ‘calcium-handling’ is largely controlled by the interplay of two membranous organelles, the transverse-axial tubule system (TATS) and the sarcoplasmic reticulum (SR) [4]. Following an action potential, the voltage gated-L-type calcium channels (LTCCs) situated mainly within the TATS membrane conduct calcium into the cleft between this organelle and the SR [3,5]. This increase in calcium concentration induces calcium-induced calcium release (CICR) from the ryanodine receptors. The elevation of calcium concentration stimulates the movement of the motor proteins and contracts the cell. This process is then reversed by the rapid re-uptake of calcium back into the SR by the enzyme SERCA2A, allowing the cell to relax. This process is known as excitation-contraction coupling (ECC) [6].

**Figure 1.**
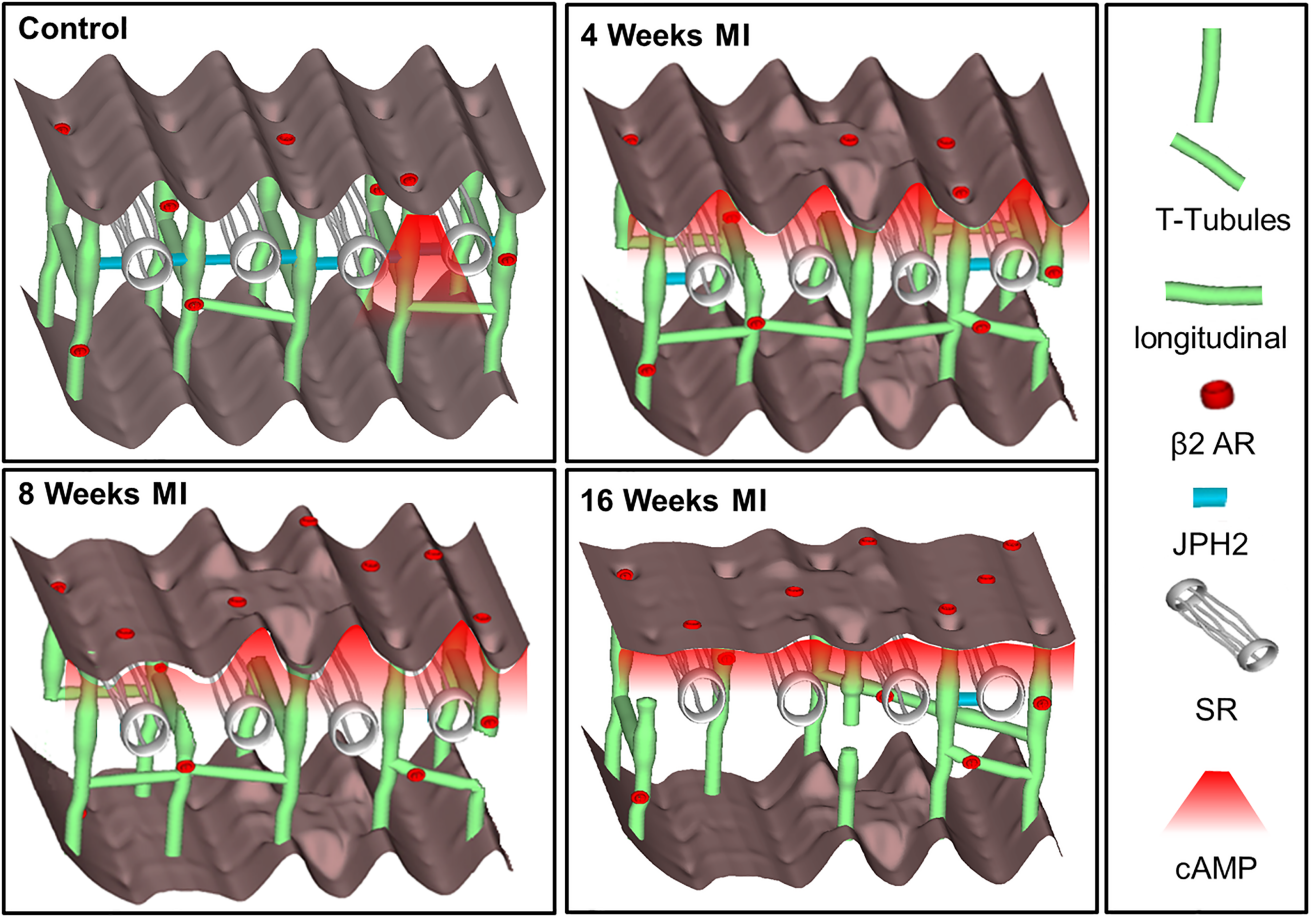
Diagram detailing the structural changes within cardiomyocytes associated with the progression from a healthy state to late stage heart failure. In the healthy (control) cells the sarcolemma presents a highly organised topography, with regularly distributed invaginations (TTs). TT β2AR generate a highly restricted pool of cAMP upon activation. In the situation of pathology, structural changes result in receptor redistribution, TT uncoupling from SR and loss of cAMP compartmentation. (Reproduced from Schobesberger et al. [44]).

Adult cardiomyocytes, especially ventricular cardiomyocytes present an extensive TAT system [7]. The transverse element of the TAT is formed by regular invaginations of the external cardiomyocyte membrane, which are linked inside the cells via axial tubules also referred to as longitudinal elements (see Figure 1) [8]. The major function of TAT is to ensure the proximity of the external membrane, SR and their respective receptor/effector panels. Caveolae are 50–100 nm diameter organelles which associate with the TAT and external membranes and incorporate specific sets of receptors and ion channels [9,10]. In neonatal cardiomyocytes and cells without an extensive TAT system caveolae assume some of the role of T-tubules [11]. Finally, the mitochondria are also significantly involved with aspects of calcium and cAMP compartmentation in cardiomyocytes [12–16].

Cardiac performance can be adapted to physiological requirements by the activation of beta-adrenergic receptors (β-AR) [17]. β-AR are members of the G-protein coupled receptor (GPCR) family and are stimulated by catecholamines *in vivo*. β1-AR and β2-AR are classical Gα_s_ coupled GPCR. When bound by a catecholamine agonist Gα_s_ dissociates from the receptor and activate adenylate cyclase (AC) producing cAMP activating the cAMP-dependent protein kinase A (PKA). PKA modulates cardiac output by phosphorylating ECC proteins. PKA phosphorylates LTCC in T-tubules and phospholamban (PLB) on the surface of SR. This results in enhanced CICR which overall has a positive inotropic and lusitropic effect on cardiac performance. Furthermore, the same event also induces the phosphorylation of the troponin I (TPNI) complex resulting in decreased myofilament sensitivity to Ca^2+^ and restricts the inotropic effect [18].

## Aspects of organellar signal compartmentation within adult cardiomyocytes

### Molecular localisation

The localisation of receptor and effector molecules is the most fundamental aspect of compartmentation processes. The organisation of specific molecular arrays within the TAT can be illustrated by the distinct functional localisation of β1-AR and β2-AR [19]. In healthy adult ventricular cardiomyocytes β2-AR are exclusively localised within T-tubules and upon activation generate spatially confined cAMP pools. The cAMP pool generated following β1-AR stimulation appears to be far-reaching and less compartmentalised. Equally, β2-AR are associated with caveolae and the scaffolding protein caveolin-3 (Cav3) [9]. Similar, to β-AR-cAMP signalling, natriuretic peptide receptor (NRP)-cGMP signalling is also compartmentalised by the TATS [20]. Two major NRP isoforms NRP1 and NRP2 activated by atrial (ANP) and brain natriuretic peptide (BNP) and C-type natriuretic peptide (CNP), respectively [21]. Activation of both isoforms appears to exhibit a cardioprotective effect by inducing an anti-arrhythmic and anti-fibrotic effect in hypertrophic cells [22]. The NRP1 is localised in TT [20] and activation results in a negative inotropic effect on the cells [23]. Like β1-AR, NRP2 is uniformly expressed throughout the plasma membrane and upon activation produce a far-reaching pool of cGMP [20]. Activation of NRP2 induces both positively lusitropic and negatively inotropic effects on murine ventricular cells [24,25]. NRP1 is like β2-AR as upon activation the cGMP pools produced are locally confined by an activity of PDE2 [20].

### Passive mechanisms

The TAT and caveolae are organelles produced by the scaffolding of membranes by structural proteins which produce physical domains [26] which act to conduct signals at the relevant sites within the cell. Equally, TAT, caveolae and other organelles provide scaffolding which allow efficient molecular interaction within the chaotic milieu of the cellular interior. The axial components of the TATS appear to handle calcium somewhat differently to the transverse elements [6]. The reversal of cardiomyocyte contractility requires the re-uptake of calcium into the SR or the mitochondria (by SERCA2A or the Mito-Ca^2+^ channel), causing the motor proteins to relax [3]. The scaffolding of molecules of PKA around in TATS and other organelles by A-kinase anchoring proteins (AKAPs) allows the rapid phosphorylation of ECC actors [2]. But it also seems to offer the passive compartmentation of cAMP by PKA buffering [26]. Essentially, the free cAMP is instantaneously bound up by PKA regulatory sub-units and the stoichiometry of PKA vs ECC molecules means that no physiological effect is exerted.

### Active mechanisms

Organelles incorporate molecules and their effectors to permit large local concentration gradients and therefore specific physiological effects. Within cardiomyocytes ACs, LTCC and β2-AR are specifically localised in TAT [26–28]. Caveolae also incorporate a specific panel of molecules including LTCC which specifically associate with CaMKII [10,29]. These molecular associations allow the activation of the signal to modulate inotropic processes. The definitive control of cAMP is performed by phosphodiesterases (PDEs), to destroy cAMP which is otherwise almost infinitely stable [19]. Inhibition of PDE5 selectively attenuates β2-AR induced contraction and Ca^2+^ transient in adult mouse ventricular cardiomyocytes. This effect was attributed to cGMP mediated activation of PDE2, which restricted β2-AR provoked cAMP pools [30]. We suggest that caveolae and other structural microdomains incorporate these molecules near to the site where the signal process is initiated and relevant effector molecules, to exert tight control on these processes. Phosphatases are also reported to be incorporated in caveolar and cholesterol-dependent microdomains [31]. The proximity of these enzymes and ECC effector molecules within the organelle ensures that phosphate groups are rapidly and efficiently removed to produce cardiomyocyte lusitropy reversing the PKA effect. Calcium and cAMP are produced in large quantities that are freely diffusible throughout the cell. However, controversy exists regarding the diffusibility of cAMP in situations of physiological relevance. PDE's can conduct cAMP by setting up directional concentration gradients by forming sinks [32]. As well as the processes of calcium transport into the cell, the Na+/Ca^2+^-exchanger can extrude calcium from the interior of the cardiomyocyte to restore normal calcium concentrations [33].

## The production and deployment of compartmentation within the myocardium

**Figure 2.**
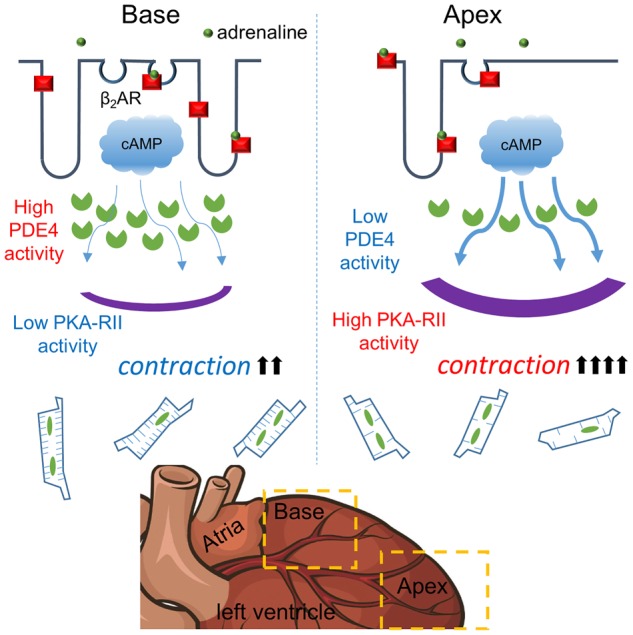
Diagram demonstrating the variability of cardiomyocyte membrane organisation depending on localisation within the LV (apical or basal myocardial regions). Within the basal region, LV cardiomyocytes present a highly organised structure, which is associated with restricted cAMP diffusion, while within the apex cardiomyocytes cAMP produced upon β2AR activation is more far-reaching. In apical cardiomyocytes β2AR can enhance cellular contractility but in basal cells cAMP is restricted by greater compartmentation. (Reproduced from Wright et al. [41]).

Neonatal cardiomyocytes are a convenient system in which to study signal compartmentation in the absence of a mature TAT system. cAMP is still extensively compartmentalised within these cells, predominantly through the action of PDEs [34]. Although clear regional inhomogeneities exist even after treatment with the non-selective phosphodiesterase inhibitor IBMX suggesting a role for passive compartmentation processes. Also, in contrast with ventricular cells, atrial myocytes from large animals such as dogs have a sparse TAT system, while atrial cells from small animals such as rats were assumed to lack TAT completely, although this has been challenged [8]. It appears that in the canine heart there are twice the number of cardiomyocytes containing TT in right atrium (RA), compared with left atrium (LA) [35]. The regional differences in sarcolemma organisation are associated with variable physiological needs [36]. The TAT system develops within the maturing cardiomyocyte concordantly with the hypertrophy of the cell. It seems that the expression of proteins such as T-Cap [37], junctophilin-2 [38] and caveolin-3 [39] allow the production of the TATS by scaffolding tubular elements. Unfortunately, controversy remains regarding the exact nature of the connection between cell size and the configuration of the TATS. The current dogma is that a larger cell demands a more extensive tubular system to function which sits uneasily with data concerning species differences, the loss of T-tubules in compensatory hypertrophy in disease and the lack of correlation between atrial cardiomyocyte size and TAT density (when excluding very small cells) [40]. We demonstrated intra- chamber variability in LV cardiomyocyte TAT organisation in adult rat cells [41]. Cells derived from the apical LV increased their contractility following β2-AR activation. Within basal cells β2-AR activity in contractility was prevented by PDE4 activity. We established that both T-tubule (TT) regularity and caveolar number were lower in apical cells despite being a similar size to basal cells (see Figure 2).

Mechanical load appears to modulate the TATS and signal compartmentation. Partially unloading the healthy heart using a heterotopic transplant model reduces the regularity of the TATS and modifies CICR, full unloading results in the loss of TAT density [42]. This suggests that the workload of the cell and the microenvironment is significantly important in patterning the relevant TATS of the cardiomyocyte. An increase in LV wall stress is reported within proximal regions of an infarct. Cardiomyocytes at infarct border zone presented decreased expression of junctophilin-2, which was associated with dysregulated TATS and decreased tubular density [36].

### Pathology

In situations of pathology, the ventricular myocytes must adapt to produce more contractile force. Under these conditions the TAT undergoes extensive remodelling [43]. In the rat heart structural and signalling changes are already observed at 4 weeks following myocardial infarction (MI) (Figure 1). These changes are characterised by reduced TT regularity and density attributed to decrease in junctophilin-2 expression [44]. At 2 months post-MI the redistribution of β2-AR was indicated by cAMP pools generated at both TT and crest regions. Finally, 4-months post-MI the β2-AR induced cAMP response was significantly decreased in the cell. The localisation and regularity of RyR component of ECC does not appear to change in HF [45]. The LTCC relocate to an extradyadic/crest space in the HF and present an abnormally high open probability [46]. It is unclear whether these abnormally localised LTCC are sparsely localised within crest regions or are associated with other molecular complexes.

At end-stage HF the TAT is almost completely lost which impairs communication between LTCC and RyR. Furthermore, sarcolemma remodelling in HF is also associated with Ca^2+^ cycling alternations, triggering arrhythmogenic events [47]. In healthy cells targeted inhibition of caveolae localised LTCCs has no overall effect on cell contractility [48]. However, in atrial cells Ca^2+^ current generated by the activity of LTCC within caveolar regions was recently proposed to contribute to pathological hypertrophy [49]. This represents the pathological decompartmentation of signalling molecules. A study of the functional implications of β-AR spatial localisation indicated that in healthy murine ventricular cardiomyocytes, β1-ARs are distributed across sarcolemma and generate far-reaching cAMP signals upon activation. The β2-AR-mediated cAMP pools are restricted to TT and produced locally confined signals. In HF the compartmentation of β2-AR-cAMP pool is lost, which is characterised by the diffusion of a secondary messenger and attributed to the redistribution of receptors to crest regions [26].

Under pathological conditions, PDE3 is substituted by PDE2 activity [50]. This results in nanodomain reorganisation and compromised cAMP/cGMP communication characterised by NPR-cGMP- dependent potentiation of β-AR-induced contractility [23]. Partial mechanical unloading (PMU) via deployment of left ventricular assisted device (LVAD) is widely implemented for HF patients [51].

Although, instinctively the opposite of the overload induced by heart failure, unloading causes effects which are somewhat similar. Cells isolated from healthy rat hearts subjected to PMU present a similar TAT phenotype to HF cells. This effect was attributed to TAT system dysregulation, resulting in an increased frequency and altered morphology of Ca^2+^ sparks [42]. Further studies have indicated that PMU significantly reduced the β2AR-induced cAMP response in the cytosolic, but not RII-PKA compartment [52]. TAT configuration is therefore dynamically tuned to a changing microenvironment rather than a static property of a specific physiological state.

## Methods for the study of signal compartmentation

In the past two decades, the scientific community has gained a more extensive understanding of signal compartmentation. This has been contributed to significantly by live-cell imaging using molecular biosensors which employ fluorescent indication and Förster resonance energy transfer (FRET). The concept of structural compartmentation is an idea as old as microscopy itself. Buxton and Brunton's seminal work from the 1980s proposed compartmentation as an explanation for the differential effects of prostacyclin and isoprenaline on heart tissue despite both enhancing cAMP production [53]. But direct evidence of the compartmentation and spatial restriction of secondary messengers is perhaps only a couple of decades old. The cardiovascular system and cardiomyocytes remain the model systems when studying this phenomenon.

### Imaging Ca^2+^ compartmentation

The first generation of fluorescent Ca^2+^ sensors were developed by Roger Tsien's research team. Amongst those the Fura2 and later Fluo-2, which are still widely used. Upon Ca^2+^ binding the florescence intensity of these molecules is increased 30-fold [54,55]. The same group has generated the yellow chameleon sensor based on a variant of green fluorescent protein (GFP) and calmodulin [56]. These reporters are not spatially localised, but when combined with confocal microscopy Ca^2+^ sparks and the spatiotemporal propagation of Ca^2+^ wave can be studied. An early study showed that in healthy rat ventricular myocytes, the Ca^2+^ transient is spatially synchronous along the transverse axis, indicating synchronous Ca^2+^ release at cell periphery and centre. The de- tubulation of cardiomyocytes via osmotic shock changed the morphology of the Ca^2+^ transient and during cell stimulation with caffeine, Ca^2+^ waves did not propagate to the cell centre [4]. These waves were later classified as V- or U-shaped and represent the activity of separate membrane associated signal compartments. The combination of super-resolution scanning ion conductance microscopy (SICM) and patch-clamp permits the resolution of tubular regions and the specific analysis of calcium channel activity within these regions. This technique has been termed super-resolution scanning patch clamp [27,46].

### FRET reporters

FRET is a phenomenon, in which energy is transferred non-radiatively from a high energy donor moiety to a lower energy acceptor causing it to fluoresce. It is possible to create molecular biosensors which utilise this effect and to genetically encode them. The most widely utilised FRET-pair are cyan and yellow fluorescent protein (CFP/YFP). Sensors are constructed by sandwiching a binding region of interest between the FRET pair. When the molecule of interest binds the sensor molecule a conformational change is produced. FRET is contingent upon the proximity of the FRET acceptor/donor pair it is exquisitely sensitive to conformational shifts. As a result, a sensor can be produced in which a binding event shifts the CFP/YFP pair away from each other to reduce FRET or shift them together to produce it [57]. In this way, the shift in FRET can be utilised as a semi-quantitative method to measure signalling events. The final feature of these sensors is that molecular localisation signals can be added to the sensor to provide readouts of signalling events in specific cellular sub-domains as we will discuss. The first FRET reporter capable of monitoring cAMP fluctuations consisted of rhodamine and fluorescein bound to regulatory and catalytic sub-units of PKA [58]. A decade later novel FRET based sensors were generated which used GFP mutants and blue GFP variant fused to catalytic and type II regulatory (RII) sub-units of PKA, respectively, [59] targeting them to cellular sub-domains incorporating type 1/2 regulatory (RI/RII). These sensors indicated the differential targeting of cAMP signals induced by isoprenaline of prostacyclin within cardiomyocytes [60]. Zaccolo's research group later generated cAMP Universal Tag for imaging experiments (CUTie) reporters. These sensors were created to provide FRET readouts in different cellular microdomains using the smallest possible cyclic nucleotide binding sub-domain (CNBD) of RII PKA linked with a targeting sequence [2]. This prevents discrepancies introduced by the steric effects caused by the interaction of large CNBDs and targeting domains. Another family of FRET reporters is based upon the CNBD of Epac molecules [13,61,62]. Transgenic mouse models have generated, with near ubiquitous expression of a cytosolic cAMP and cGMP sensor [63,64]. Nikolaev's research group have generated plasma membrane bound Epac (pmEpac2) and phospholamban targeted Epac (Epac- PLN) sensors. More recently a novel Epac1-camps based reporter linked to PLM (PLM-Epac1) localised to the vicinity of NKA channels investigated cAMP dynamics was developed [50] to study cAMP compartmentalisation within the vicinity of a specific ion channel. The results of this study indicated that within healthy ventricular cardiomyocytes, cAMP pools generated within this domain are tightly regulated by PDE3 activity. Sensors based on A-kinase anchoring region (AKAR) have been produced to measure PKA activity [65,66]. These sensors showed that β1-AR, but not β2-AR induces nuclear response in rat cardiomyocytes. It appears that the activity of PDE4 anchored to the nuclear membrane by mAKAPβ prevents activation of nuclear PKA by β2-AR-cAMP [66].

### SICM/FRET

SICM overcomes the resolution limit of conventional light microscopy and provides 3D topographical images of cell membranes. A glass pipette with an electrode inside is moved toward the surface of interest. If the pipette encounters a structure the conductance within the circuit will drop. The pipette is moved in the *xy* direction across the sample of interest to build up a conductance map of the surface which can generally be considered as a representation of topography. In a modification of this method, the *z*-resolution of the technique can be enhanced by hopping the probe along the surface at predefined intervals. The inner diameter of the scanning glass probe determines the resolution of SICM, which can currently achieve a lateral resolution of ∼10 nm [67]. The SICM image is built up without interaction between the probe and the sample meaning that it is both non-destructive and non-contact. Furthermore SICM also permits localised molecule delivery through the nanopipette by application of pressure to push a hydrojet from the pipette, or via modulation of the current to produce electrophoresis [68–71]. The combination of SICM and FRET microscopy has given researchers an invaluable understanding of β-AR subtype distribution within cellular domains in health and disease [26]. SICM/FRET permits the evaluation of molecular events within the FRET functionalised cell in response to the stimulation of highly localised membrane regions such as the t-tubules of cardiomyocytes.

## Conclusion

Signal compartmentation is essential for the control of cardiac activity and we have only just begun to uncover the underlying mechanisms of these processes. This is also true of the changes induced in pathological conditions, such as hypertrophy and HF. Restoration of normal signal compartmentation is essential for the mitigation of disease and the regeneration of cardiac function. We speculate that therapeutics which attempt to remedy the loss of calcium and cAMP will be crucial to the fight against heart disease. This review provides a brief summary of this area of study and techniques that are currently available for the study of signal compartmentation.

## Perspectives

- The compartmentation of calcium and cAMP is crucial to the normal function of the cardiomyocyte as they allow the cell to respond to external stimuli with specificity.
- The loss of signal compartmentation is a consistent component of different forms of heart failure. The degree of organisation of signalling domains within the cardiomyocytes is extremely subtle and probably occurs on the order of nanometres. Fluorescent and FRET-based sensors and probe microscopy are beginning to dissect these mechanisms.
- Therapeutics which can restore normal signal compartmentation are necessary to combat the burden of heart failure on a global scale.

## Open Access

Open access for this article was enabled by the participation of Imperial College London in an all-inclusive Read & Publish pilot with Portland Press and the Biochemical Society under a transformative agreement with JISC.

## Abbreviations

β-AR: beta-adrenergic receptors
ACs: adenylate cyclases
AKAPs: A-kinase anchoring proteins
AKAR: A-kinase anchoring region
BNP: brain natriuretic peptide
CICR: calcium-induced calcium release
CNBD: cyclic nucleotide binding sub-domain
CNP: C-type natriuretic peptide
ECC: excitation-contraction coupling
FRET: Förster resonance energy transfer
GFP: green fluorescent protein
GPCR: G-protein coupled receptor
LTCCs: L-type calcium channels
LVAD: left ventricular assisted device
MI: myocardial infarction
NRP: natriuretic peptide receptor
PDEs: phosphodiesterases
PKA: protein kinase A
PMU: Partial mechanical unloading
SICM: scanning ion conductance microscopy
SR: sarcoplasmic reticulum
TATS: transverse-axial tubule system

## References

[1] Wright P.T., Schobesberger S. and Gorelik J. (2015) Studying GPCR/cAMP pharmacology from the perspective of cellular structure. Front. Pharmacol. 6, 148 10.3389/fphar.2015.00148 26236239 PMC4505077

[2] Surdo N.C., Berrera M., Koschinski A., Brescia M., MacHado M.R., Carr C. et al. (2017) FRET biosensor uncovers cAMP nano-domains at b-adrenergic targets that dictate precise tuning of cardiac contractility. Nat. Commun. 8, 15031 10.1038/ncomms15031 28425435 PMC5411486

[3] Bers D.M. (2002) Cardiac excitation-contraction coupling. Nature 415, 198–205 10.1038/415198a 11805843

[4] Brette F., Despa S., Bers D.M. and Orchard C.H. (2005) Spatiotemporal characteristics of SR Ca^2+^ uptake and release in detubulated rat ventricular myocytes. J. Mol. Cell. Cardiol. 39, 804–812 10.1016/j.yjmcc.2005.08.005 16198369

[5] Cheng H., Cannell M.B. and Lederer W.J. (1994) Propagation of excitation-contraction coupling into ventricular myocytes. Pflugers Arch. 428, 415–417 10.1007/BF00724526 7816564

[6] Wuytack F., Raeymaekers L. and Missiaen L. (2002) Molecular physiology of the SERCA and SPCA pumps. Cell Calcium 32, 279–305 10.1016/S0143416002001847 12543090

[7] Crossman D.J., Young A.A., Ruygrok P.N., Nason G.P., Baddelely D., Soeller C. et al. (2015) T-tubule disease: relationship between t-tubule organization and regional contractile performance in human dilated cardiomyopathy. J. Mol. Cell. Cardiol. 84, 170–178 10.1016/j.yjmcc.2015.04.022 25953258 PMC4467993

[8] Brandenburg S., Kohl T., Williams G.S.B., Gusev K., Wagner E., Rog-Zielinska E.A. et al. (2016) Axial tubule junctions control rapid calcium signaling in atria. J. Clin. Invest. 126, 3999–4015 10.1172/JCI88241 27643434 PMC5096811

[9] Wright P.T., Nikolaev V.O., O'Hara T., Diakonov I., Bhargava A., Tokar S. et al. (2014) Caveolin-3 regulates compartmentation of cardiomyocyte beta2-adrenergic receptor-mediated cAMP signaling. J. Mol. Cell. Cardiol. 67, 38–48 10.1016/j.yjmcc.2013.12.003 24345421 PMC4266930

[10] Best J.M. and Kamp T.J. (2012) Different subcellular populations of L-type Ca^2+^ channels exhibit unique regulation and functional roles in cardiomyocytes. J. Mol. Cell. Cardiol. 52, 376–387 10.1016/j.yjmcc.2011.08.014 21888911 PMC3264751

[11] Rybin V.O., Pak E., Alcott S. and Steinberg S.F. (2003) Developmental changes in β2-adrenergic receptor signaling in ventricular myocytes: the role of Gi proteins and caveolae microdomains. Mol. Pharmacol. 63, 1338–1348 10.1124/mol.63.6.1338 12761344

[12] Miragoli M., Sanchez-Alonso J.L., Bhargava A., Wright P.T., Sikkel M., Schobesberger S. et al. (2016) Microtubule-dependent mitochondria alignment regulates calcium release in response to nanomechanical stimulus in heart myocytes. Cell Rep. 14, 1338–1348 10.1016/j.celrep.2015.12.014 PMC4983655 26725114

[13] DiPilato L.M., Cheng X. and Zhang J. (2004) Fluorescent indicators of cAMP and Epac activation reveal differential dynamics of cAMP signaling within discrete subcellular compartments. Proc. Natl Acad. Sci. U.S.A. 101, 16513–8 10.1073/pnas.0405973101 15545605 PMC534508

[14] DiPilato L.M. and Zhang J. (2009) The role of membrane microdomains in shaping beta2-adrenergic receptor-mediated cAMP dynamics. Mol. Biosyst. 5, 832–837 10.1039/b823243a 19603118

[15] Llopis J., McCaffery J.M., Miyawaki A., Farquhar M.G. and Tsien R.Y. (1998) Measurement of cytosolic, mitochondrial, and Golgi pH in single living cells with green fluorescent proteins. Proc. Natl Acad. Sci. U.S.A. 95, 6803–6808 10.1073/pnas.95.12.6803 9618493 PMC22642

[16] Di Benedetto G., Scalzotto E., Mongillo M. and Pozzan T. (2013) Mitochondrial Ca^2+^ uptake induces cyclic AMP generation in the matrix and modulates organelle ATP levels. Cell Metab. 17, 965–975 10.1016/j.cmet.2013.05.003 23747252

[17] Zaglia T., Milan G., Franzoso M., Bertaggia E., Pianca N., Piasentini E. et al. (2013) Cardiac sympathetic neurons provide trophic signal to the heart via β2-adrenoceptor-dependent regulation of proteolysis. Cardiovasc. Res. 97, 240–250 10.1093/cvr/cvs320 23090606

[18] Xiao R.P. and Lakatta E.G. (1993) Beta 1-adrenoceptor stimulation and beta 2-adrenoceptor stimulation differ in their effects on contraction, cytosolic Ca^2+^, and Ca^2+^ current in single rat ventricular cells. Circ. Res. 73, 286–300 10.1161/01.RES.73.2.286 8101141

[19] Nikolaev V.O., Bünemann M., Schmitteckert E., Lohse M.J. and Engelhardt S. (2006) Cyclic AMP imaging in adult cardiac myocytes reveals far-reaching β1-adrenergic but locally confined β2-adrenergic receptor–mediated signaling. Circ Res. 99, 1084–1091 10.1161/01.RES.0000250046.69918.d5 17038640

[20] Subramanian H., Froese A., Jönsson P., Schmidt H., Gorelik J. and Nikolaev V.O. (2018) Distinct submembrane localisation compartmentalises cardiac NPR1 and NPR2 signalling to cGMP. Nat. Commun. 9, 2446 10.1038/s41467-018-04891-5 29934640 PMC6014982

[21] Potter L.R., Abbey-Hosch S. and Dickey D.M. (2006) Natriuretic peptides, their receptors, and cyclic guanosine monophosphate-dependent signaling functions. Endocr. Rev. 27, 47–72 10.1210/er.2005-0014 16291870

[22] Ennis I.L., Garciarena C.D., Escudero E.M., Pérez N.G., Dulce R.A., Camilión de Hurtado M.C. et al. (2007) Normalization of the calcineurin pathway underlies the regression of hypertensive hypertrophy induced by Na^+^/H^+^ exchanger-1 (NHE-1) inhibition. Can. J. Physiol. Pharmacol. 85, 301–310 10.1139/y06-072 17612638

[23] Perera R.K., Sprenger J.U., Steinbrecher J.H., Hübscher D., Lehnart S.E., Abesser M. et al. (2015) Microdomain switch of cGMP-regulated phosphodiesterases leads to ANP-induced augmentation of beta-adrenoceptor-stimulated contractility in early cardiac hypertrophy. Circ. Res. 116, 1304–1311 10.1161/CIRCRESAHA.116.306082 25688144

[24] Moltzau L.R., Aronsen J.M., Meier S., Skogestad J., Ørstavik Ø., Lothe G.B. et al. (2014) Different compartmentation of responses to brain natriuretic peptide and C-type natriuretic peptide in failing rat ventricle. J. Pharmacol. Exp. Ther. 350, 681–690 10.1124/jpet.114.214882 25022512

[25] Pierkes M., Gambaryan S., Boknik P., Lohmann S.M., Schmitz W., Potthast R. et al. (2002) Increased effects of C-type natriuretic peptide on cardiac ventricular contractility and relaxation in guanylyl cyclase A-deficient mice. Cardiovasc. Res. 53, 852–861 10.1016/S0008-6363(01)00543-0 11922895

[26] Nikolaev V.O., Moshkov A., Lyon A.R., Miragoli M., Novak P., Paur H. et al. (2010) β2-adrenergic receptor redistribution in heart failure changes cAMP compartmentation. Science 327, 1653–1657 10.1126/science.1185988 20185685

[27] Bhargava A., Lin X., Novak P., Mehta K., Korchev Y., Delmar M. et al. (2013) Super-resolution scanning patch clamp reveals clustering of functional ion channels in adult ventricular myocyte. Circ. Res. 112, 1112–1120 10.1161/CIRCRESAHA.111.300445 23438901 PMC3899650

[28] Laflamme M.A. and Becker P.L. (1999) Gs and adenylyl cyclase in transverse tubules of heart: implications for cAMP-dependent signaling. Am. J. Physiol. Circ. Physiol. 277, H1841–H1848 10.1152/ajpheart.1999.277.5.H1841 10564138

[29] Head B.P., Patel H.H., Roth D.M., Lai N.C., Niesman I.R., Farquhar M.G. et al. (2005) G-protein-coupled receptor signaling components localize in both sarcolemmal and intracellular caveolin-3- associated microdomains in adult cardiac myocytes. J. Biol. Chem. 280, 31036–31044 10.1074/jbc.M502540200 15961389

[30] Isidori A.M., Cornacchione M., Barbagallo F., Di Grazia A., Barrios F., Fassina L. et al. (2015) Inhibition of type 5 phosphodiesterase counteracts beta2-adrenergic signalling in beating cardiomyocytes. Cardiovasc. Res. 106, 408–420 10.1093/cvr/cvv123 25852085

[31] MacDougall D.A., Agarwal S.R., Stopford E.A., Chu H., Collins J.A., Longster A.L. et al. (2012) Caveolae compartmentalise β2-adrenoceptor signals by curtailing cAMP production and maintaining phosphatase activity in the sarcoplasmic reticulum of the adult ventricular myocyte. J. Mol. Cell. Cardiol. 52, 388–400 10.1016/j.yjmcc.2011.06.014 21740911 PMC3270222

[32] Bedioune I., Bobin P., Leroy J., Fischmeister R. and Vandecasteele G. (2017) Cyclic nucleotide phosphodiesterases and compartmentation in normal and diseased heart In Microdomains in the Cardiovascular System (Nikolaev V. and Zaccolo M., eds), pp. 97–116, Springer International Publishing, Cham

[33] Ke H.-Y., Yang H.-Y., Francis A.J., Collins T.P., Surendran H., Alvarez-Laviada A. et al. (2019) Changes in cellular Ca^2+^ and Na^+^ regulation during the progression towards heart failure in the guinea pig. J. Physiol. 10.1113/JP277038 PMC7187457 30811606

[34] Zaccolo M. and Pozzan T. (2002) Discrete microdomains with high concentration of cAMP in stimulated rat neonatal cardiac myocytes. Science 295, 1711–1715 10.1126/science.1069982 11872839

[35] Arora R., Aistrup G.L., Supple S., Frank C., Singh J., Tai S. et al. (2017) Regional distribution of T-tubule density in left and right atria in dogs. Heart Rhythm. 14, 273–281 10.1016/j.hrthm.2016.09.022 27670628 PMC5484147

[36] Frisk M., Ruud M., Espe E.K.S., Aronsen J.M., Røe Å.T, Zhang L. et al. (2016) Elevated ventricular wall stress disrupts cardiomyocyte t-tubule structure and calcium homeostasis. Cardiovasc. Res. 112, 443–451 10.1093/cvr/cvw111 27226008 PMC5031949

[37] Hong T.-T., Smyth J.W., Gao D., Chu K.Y., Vogan J.M., Fong T.S. et al. (2010) BIN1 localizes the L-type calcium channel to cardiac T-tubules. PLoS Biol. 8, e1000312 10.1371/journal.pbio.1000312 20169111 PMC2821894

[38] Beavers D.L., Landstrom A.P., Chiang D.Y. and Wehrens X.H.T. (2014) Emerging roles of junctophilin-2 in the heart and implications for cardiac diseases. Cardiovasc. Res. 103, 198–205 10.1093/cvr/cvu151 24935431 PMC4809974

[39] Wong J., Baddeley D., Bushong E.A., Yu Z., Ellisman M.H., Hoshijima M. et al. (2013) Nanoscale distribution of ryanodine receptors and caveolin-3 in mouse ventricular myocytes: dilation of T-tubules near junctions. Biophys. J. 104, L22–L24 10.1016/j.bpj.2013.02.059 23746531 PMC3672889

[40] Yue X., Zhang R., Kim B., Ma A., Philipson K.D. and Goldhaber J.I. (2017) Heterogeneity of transverse-axial tubule system in mouse atria: remodeling in atrial-specific Na^+^–Ca^2+^ exchanger knockout mice. J. Mol. Cell. Cardiol. 108, 50–60 10.1016/j.yjmcc.2017.05.008 28529049 PMC5791526

[41] Wright P.T., Bhogal N.K., Diakonov I., Pannell L.M.K., Perera R.K., Bork N.I. et al. (2018) Cardiomyocyte membrane structure and cAMP compartmentation produce anatomical variation in β2AR- cAMP responsiveness in murine hearts. Cell Rep. 23, 459–469 10.1016/j.celrep.2018.03.053 29642004 PMC5912947

[42] Ibrahim M., Kukadia P., Siedlecka U., Cartledge J.E., Navaratnarajah M., Tokar S. et al. (2012) Cardiomyocyte Ca^2+^ handling and structure is regulated by degree and duration of mechanical load variation. J. Cell. Mol. Med. 16, 2910–2918 10.1111/j.1582-4934.2012.01611.x 22862818 PMC4393719

[43] Lyon A.R., Nikolaev V.O., Miragoli M., Sikkel M.B., Paur H., Benard L. et al. (2012) Plasticity of surface structures and β2-adrenergic receptor localization in failing ventricular cardiomyocytes during recovery from heart failure. Circ. Heart Fail. 5, 357–365 10.1161/CIRCHEARTFAILURE.111.964692 22456061 PMC4886822

[44] Schobesberger S., Wright P., Tokar S., Bhargava A., Mansfield C., Glukhov A.V. et al. (2017) T-tubule remodelling disturbs localized β2-adrenergic signalling in rat ventricular myocytes during the progression of heart failure. Cardiovasc. Res. 113, 770–782 10.1093/cvr/cvx074 28505272 PMC5437368

[45] Bryant S.M., Kong C.H.T., Watson J., Cannell M.B., James A.F. and Orchard C.H. (2015) Altered distribution of ICa impairs Ca release at the t-tubules of ventricular myocytes from failing hearts. J. Mol. Cell. Cardiol. 86, 23–31 10.1016/j.yjmcc.2015.06.012 26103619 PMC4564288

[46] Sanchez-Alonso J.L., Bhargava A., O'Hara T., Glukhov A V., Schobesberger S., Bhogal N.K. et al. (2016) Microdomain-specific modulation of L-type calcium channels leads to triggered ventricular arrhythmia in heart failure. Circ. Res. 119, 944–955 10.1161/CIRCRESAHA.116.308698 27572487 PMC5045818

[47] Nivala M., Song Z., Weiss J.N. and Qu Z. (2015) T-tubule disruption promotes calcium alternans in failing ventricular myocytes: mechanistic insights from computational modeling. J. Mol. Cell. Cardiol. 79, 32–41 10.1016/j.yjmcc.2014.10.018 25450613 PMC4323351

[48] Makarewich C.A., Correll R.N., Gao H., Zhang H., Yang B., Berretta R.M. et al. (2012) A caveolae-targeted L-type Ca^2+^ channel antagonist inhibits hypertrophic signaling without reducing cardiac contractility. Circ. Res. 110, 669–674 10.1161/CIRCRESAHA.111.264028 22302787 PMC3324037

[49] Balycheva M., Faggian G., Glukhov A V. and Gorelik J. (2015) Microdomain–specific localization of functional ion channels in cardiomyocytes: an emerging concept of local regulation and remodelling. Biophys. Rev. 7, 43–62 10.1007/s12551-014-0159-x 28509981 PMC5425752

[50] Bastug-Özel Z., Wright P.T., Kraft A.E., Pavlovic D., Howie J., Froese A. et al. (2018) Heart failure leads to altered β2-adrenoceptor/cyclic adenosine monophosphate dynamics in the sarcolemmal phospholemman/Na,K ATPase microdomain. Cardiovasc. Res. 115, 546–555 10.1093/cvr/cvy221 PMC6383061 30165515

[51] Holmberg E., Ahn H. and Peterzén B. (2017) More than 20 years’ experience of left ventricular assist device implantation at a non-transplant centre. Scand. Cardiovasc. J. 51, 293–298 10.1080/14017431.2017.1388536 29029567

[52] Wright P.T., Sanchez-Alonso J.L., Lucarelli C., Alvarez-Laviada A., Poulet C.E., Bello S.O. et al. (2018) Partial mechanical unloading of the heart disrupts L-type calcium channel and beta-adrenoceptor signaling microdomains. Front. Physiol. 9, 1302 10.3389/fphys.2018.01302 30283354 PMC6157487

[53] Buxton I.L. and Brunton L.L. (1983) Compartments of cyclic AMP and protein kinase in mammalian cardiomyocytes. J. Biol. Chem. 258, 10233–9 PMID:6309796

[54] Grynkiewicz G., Poenie M. and Tsien R.Y. (1985) A new generation of Ca^2+^ indicators with greatly improved fluorescence properties. J. Biol. Chem. 260, 3440–3450 PMID:3838314

[55] Minta A., Kao J.P. and Tsien R.Y. (1989) Fluorescent indicators for cytosolic calcium based on rhodamine and fluorescein chromophores. J. Biol. Chem. 264, 8171–8178 PMID:2498308

[56] Miyawaki A., Griesbeck O., Heim R. and Tsien R.Y. (1999) Dynamic and quantitative Ca^2+^ measurements using improved cameleons. Proc. Natl Acad. Sci. U.S.A. 96, 2135–2140 10.1073/pnas.96.5.2135 10051607 PMC26749

[57] Nikolaev V.O., Gambaryan S. and Lohse M.J. (2006) Fluorescent sensors for rapid monitoring of intracellular cGMP. Nat. Methods 3, 23–25 10.1038/nmeth816 16369548

[58] Adams S.R., Harootunian A.T., Buechler Y.J., Taylor S.S. and Tsien R.Y. (1991) Fluorescence ratio imaging of cyclic AMP in single cells. Nature 349, 694–697 10.1038/349694a0 1847505

[59] Zaccolo M., De Giorgi F., Cho C.Y., Feng L., Knapp T., Negulescu P.A. et al. (2000) A genetically encoded, fluorescent indicator for cyclic AMP in living cells. Nat. Cell Biol. 2, 25–29 10.1038/71345 10620803

[60] Di Benedetto G., Zoccarato A., Lissandron V., Terrin A., Li X., Houslay M.D. et al. (2008) Protein kinase A type I and type II define distinct intracellular signaling compartments. Circ. Res. 103, 836–844 10.1161/CIRCRESAHA.108.174813 18757829

[61] Nikolaev V.O., Bünemann M., Hein L., Hannawacker A. and Lohse M.J. (2004) Novel single chain cAMP sensors for receptor-induced signal propagation. J. Biol. Chem. 279, 37215–8 10.1074/jbc.C400302200 15231839

[62] Ponsioen B., Zhao J., Riedl J., Zwartkruis F., van der Krogt G., Zaccolo M. et al. (2004) Detecting cAMP-induced epac activation by fluorescence resonance energy transfer: Epac as a novel cAMP indicator. EMBO Rep. 5, 1176–1180 10.1038/sj.embor.7400290 15550931 PMC1299185

[63] Calebiro D., Nikolaev V.O., Gagliani M.C., de Filippis T., Dees C., Tacchetti C. et al. (2009) Persistent cAMP-signals triggered by internalized G-protein–coupled receptors. PLoS Biol. 7, e1000172 10.1371/journal.pbio.1000172 19688034 PMC2718703

[64] Gøtz K.R., Sprenger J.U., Perera R.K., Steinbrecher J.H., Lehnart S.E., Kuhn M. et al. (2014) Transgenic mice for real-time visualization of cGMP in intact adult cardiomyocytes. Circ. Res. 114, 1235–1245 10.1161/CIRCRESAHA.114.302437 24599804

[65] Allen M.D. and Zhang J. (2006) Subcellular dynamics of protein kinase A activity visualized by FRET-based reporters. Biochem. Biophys. Res. Commun. 348, 716–721 10.1016/j.bbrc.2006.07.136 16895723

[66] Bedioune I., Lefebvre F., Lechêne P., Varin A., Domergue V., Kapiloff M.S. et al. (2018) PDE4 and mAKAPbeta are nodal organizers of beta2-ARs nuclear PKA signalling in cardiac myocytes. Cardiovasc. Res. 114, 1499–1511 10.1093/cvr/cvy110 29733383 PMC6106106

[67] Shevchuk A.I., Frolenkov G.I., Sánchez D., James P.S., Freedman N., Lab M.J. et al. (2006) Imaging proteins in membranes of living cells by high-resolution scanning ion conductance microscopy. Angew. Chem. Int. Ed. 45, 2212–2216 10.1002/anie.200503915 16506257

[68] Gorelik J., Ali N.N., Shevchuk A.I., Lab M., Williamson C., Harding S.E. et al. (2006) Functional characterization of embryonic stem cell-derived cardiomyocytes using scanning ion conductance microscopy. Tissue Eng. 12, 657–664 10.1089/ten.2006.12.657 16674281

[69] Gorelik J., Shevchuk A., Ramalho M., Elliott M., Lei C., Higgins C.F. et al. (2002) Scanning surface confocal microscopy for simultaneous topographical and fluorescence imaging: application to single virus-like particle entry into a cell. Proc. Natl Acad. Sci. U.S.A. 99, 16018–16023 10.1073/pnas.252458399 12466501 PMC138557

[70] Gu Y., Gorelik J., Spohr H.A., Shevchuk A., Lab M.J., Harding S.E. et al. (2002) High-resolution scanning patch-clamp: new insights into cell function. FASEB J. 16, 748–750 10.1096/fj.01-1024fje 11923226

[71] Korchev Y.E., Gorelik J., Lab M.J., Sviderskaya E V., Johnston C.L., Coombes C.R. et al. (2000) Cell volume measurement using scanning ion conductance microscopy. Biophys. J. 78, 451–457 10.1016/S0006-3495(00)76607-0 10620308 PMC1300652

